# Systematic review: dorsal bridge plating in distal radius fractures

**DOI:** 10.1007/s12306-024-00822-4

**Published:** 2024-07-05

**Authors:** I. Drummond, M. Durand-Hill, N. Jones, P. J. O’Hagan, D. Edwards

**Affiliations:** 1 Royal London Hospital, Barts Health NHS Trust, London, UK; 2 Centre for Neuroscience, Surgery and Trauma within Blizard Institute, Queen Mary University of London, London, UK

**Keywords:** Dorsal bridge plate, Spanning bridge plate, Distraction plate, Distal radius fracture

## Abstract

**Purpose:**

Distal radius fractures are the most common upper limb fractures in adults (up to 18% of all fractures in the Emergency Department). Conservative management is possible for the majority, the preferred surgical technique being volar plate fixation. Dorsal bridge plating (DBP) is an alternative method of treatment for complex fractures. DBP acts as an internal fixator and can be used in patients needing early rehabilitation. This systematic review assesses the demographics, functional and radiological outcomes and complications of using DBP in patients with distal radius fractures compared to volar plate fixation.

**Methods:**

A literature search of PubMed, Cochrane, EMBASE and Google Scholar was performed according to PRISMA guidelines. Seven hundred and sixty-one articles were found; 11 articles met the inclusion criteria. Cadaveric studies and case studies of less than five patients were excluded. Primary outcome measures were functional and radiological outcomes. Complications were recorded as secondary outcomes.

**Results:**

Three hundred and ninety-four patients were included in the study with an average age of 54.8 years (53.9% male and 46.1% female). Weighted mean follow-up was 55.2 weeks; the mean time to plate removal was 17.3 weeks with a mean DASH score of 25.7. The weighted range of movement was 46.9° flexion, 48.8° extension, 68.4° pronation and 67.5° supination. The radiological parameters show satisfactory outcomes with a mean radial height of 10mm, volar tilt of 3.1°, ulnar variance of 0.5mm and radial inclination of 18.8°. The complication rate was 11.4%. Digital stiffness was the most common complication but improved if tenolysis was performed at plate removal.

**Conclusions:**

DBP is a good alternative to volar plating for complex distal radius fractures. The functional outcomes showed a slight loss of range of movement, whereas the radiological outcomes were within recommended limits. A significant disadvantage of the plate is the need for further surgical removal.

## Introduction

Distal radius fractures are the most common upper limb fractures and account for 17–18% of all adult fractures in the Emergency Department [1–4]. Operative techniques used to manage distal radius fractures include closed reduction, percutaneous K-wiring and internal/external fixation; of which, Volar locking plates have become the operative technique of choice [5].

This said, stability can be a concern when volar locking plates are used to manage patients with highly comminuted intra-articular fractures, additional radiocarpal ligamentous injuries, dorsal shearing fractures and those with poor bone quality [1, 5–7]. As a result, stabilisation of these fractures using an external fixator has traditionally been used for such complex injuries. This allows soft tissues to recover from the trauma and gives time for further imaging and planning, whilst reducing pain for the patient. However, these constructs are heavy, do not allow easy use of the arm perioperatively and are often complicated with pin site infections, stiffness and have the potential for fracture mal-reduction due to the pins being able to be accessed externally and knocked.

The dorsal bridge plate (DBP) was first described in 1998 [8]. The DBP has been proposed for use in polytrauma patients requiring damage control surgery or those needing to ambulate early in the post-operative period as it gives enough stability for weight bearing, irrespective of fracture configuration [9, 10]. In this way, a DBP is considered by many to be preferential to an external fixator, giving the same fracture stabilisation without the complication profile [2, 3, 8]. The plate can be left in for longer than an external fixator; however, the plate requires surgical removal after 12–16 weeks [3, 11].

There remains variation in practice amongst clinicians treating DRFs suitable for either treatment due to the limited literature available. Previous reviews have analysed functional and radiological outcomes in isolation [2]. The aim of this study is combine such studies to give a more powerful representation of the radiological and functional outcomes of DBPs. The study will also report on the complications associated with DBP in distal radius fractures compared to volar plate fixation and summarise findings in an effort to further inform and standardise clinical practice.

## Method

### Protocol and registration

The review was registered on PROSPERO prior to starting.

### Literature search and selection of studies

Following the Preferred Reporting Items for Systematic Reviews and Meta-Analyses (PRISMA) guidelines, EMBASE, PubMed, Cochrane and Google Scholar and were all searched using (((Distract*) AND (Plat*)) OR ((Bridg*) AND (Plat*))) AND (distal radius) on 1st May 2021. There was no date restriction. Bibliographies of all identified papers were reviewed to ascertain if any listed were suitable for inclusion (*n* = 761).

**Fig. 1:**
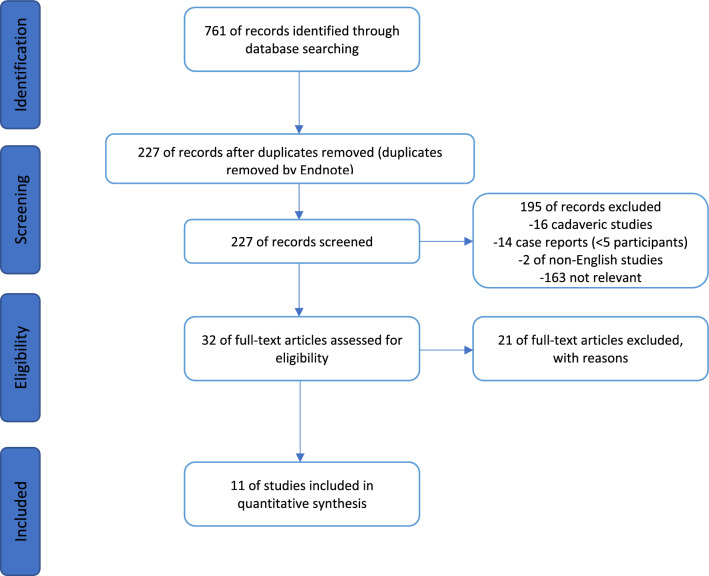
The modified PRISMA flowchart for included and excluded studies

Duplicate studies identified were removed (*n* = 534). The articles were assessed by two people. First, the titles and abstracts were screened against the inclusion criteria as shown in Table 1 for their relevance (*n* = 534). Full articles were then analysed according to the inclusion and exclusion criteria. A modified PRISMA flowchart is used to display the methodology of this search in graphic form (Fig. 1).

**Table 1:** Eligibility criteria

Inclusion criteria	Exclusion criteria
English language	Cadaveric studies
Review of the outcomes of distal radius fractures managed with a DBP	Individual case studies, or those with < 5 participants
No time or date restrictions or limits were applied to the search	
All study designs were included	
Only case studies with 5 or more participants were included in the final selection of articles

### Data extraction and outcomes

A pilot study was performed in order to create a proforma. The data collected included: study designs, demographics of the population included in the study, sample size, outcomes and complications. All the data were collated on Microsoft Excel.

The primary outcomes:

- Demographic data
- Mechanism of injury
- Functional outcomes including disabilities of the arm, shoulder and hand (DASH) scores and range of movement (ROM) including grip strength, flexion, extension, pronation, supination, ulnar deviation and radial deviation. DASH scoring system quantifies the disability with a range between 0 (no disability) and 100 (maximum disability).
- Radiological outcomes including radial height, volar tilt, ulnar variance and radial inclination

Secondary outcomes:

- Complications including infection, need for further operations, pain, tendon injuries and mal-or non-unions.

The primary and secondary outcomes were analysed in a quantitative manner. Weighted averages were calculated depending on the studies that analysed that particular outcomes. The outcomes were presented as a collective as seen in the results. Heterogeneity was evaluated to see if quantitative assessment with meta-analysis was possible.

### Data analysis

Individual studies were assessed for risk of bias using the Risk of Bias In Non-Randomised Studies of Intervention (ROBINS-I) tool. The leads to the studies being labelled ‘low risk’, ‘moderate risk’, ‘high risk’ or ‘critical risk’ based on seven different criteria.

## Results

The initial search found 761 studies, and 11 were eligible for inclusion [1, 4, 5, 12–19]. Of these, nine were retrospective [1, 4, 5, 12, 14, 15, 17–19], one was observational [16] and one was a case study of more than five participants [13]. This accounted for 394 patients.

Of those that were excluded 227 studies were duplicates, 16 were cadaveric, 14 were case reports of less than five participants, two were not in English and the remainder were not relevant. Due to the heterogeneity of data collection methods and methodology between studies, a meta-analysis was not possible. Therefore, results are presented of a quantitive nature.

### Demographics

Table 2 describes the demographics from the included studies. The weighted mean age was 52.1 with a range from 41 to 72 years. The follow-up of patients was between 19.5 and 150.8 weeks (weighted mean 55.2 weeks). The weighted mean time to plate removal was 17.3 weeks. 58.9% of the participants were male. The majority of mechanisms of injuries were high-energy falls and road traffic accidents as shown in Table 3.

**Table 2:** Demographics

Authors	Type of paper	*n*	Mean age	Sex (% of male)	Dominant hand injured (%)	Polytrauma (%)	Fracture classification	Supplementary metalwork (%)	Days to plate removal (weeks)	Follow-up time (weeks)
Bouvet et al. [17]	Retrospective	21	52.0	43.0	NR	33.3	C3	100.0	15.1	NR
Carula et al. [16]	Observational	25	NR	NR	NR	NR	C2, C3	NR	NR	NR
Dodds et al. [15]	Retrospective	25	54.6	56.0	NR	100.0	B (7); C (18)	NR	26.4	55.0
Hanel et al. [14]	Retrospective	62	47.8	63.0	60.0	37.1	A3 (18), B2 (3), C3 (41)	NR	16.0	46.8
Hanel et al. [18]	Retrospective	134	48.5	66.2	NR	64.9	NR	55.2	19.4	43.0
Huish et al. [12]	Retrospective	19	47.8	58.0	36.0	NR	NR	57.9	11.5	NR
Lauder et al. [4]	Retrospective	18	61.0	72.0	44.0	NR	C3	NR	12.0	150.8
Richard et al. [1]	Retrospective	33	70.0	30.0	NR	NR	C2, C3	21.2	17.0	47.0
Ruch et al. [19]	Retrospective	22	54.6	55.0	73.0	59.1	A3 (2); C3 (20)	NR	17.7	107.0
Sharareh et al. [5]	Retrospective	24	41.0	NR	NR	NR	C3	0.0	12.4	19.5
Tinsley and Ilyas [13]	Case series	11	72.0	NR	NR	NR	A2 (1), A3 (3), C1 (2), C2 (2), C3 (3)	NR	11.0	NR
Weighted mean		394	52.1	58.9	56.2	58.7	0	48.9	17.3	55.2

**Table 3:** Mechanism of Injury

	*n*	Low-energy fall	High-energy fall	Road traffic accident	Other
Dodds et al. [15]	25	5	15	5	0
Hanel et al. [18]	134	0	89	38	3
Huish et al. [12]	19	9	3	7	0
Richard et al. [1]	33	23	0	9	1
Ruch et al. [19]	22	1	0	16	5
Sharareh et al. [5]	24	3	14	6	1
Tinsley and Ilyas [13]	11	11	0	0	0
Weighted %		19.70	45.83	30.68	3.79

The majority of the papers subclassified the fracture pattern into AO classification. Of those that subclassified, 86% were C-type fractures, 4% were B-type and 10% were A-type. Only five studies documented if any of the fractures were open, of these, the rate of open fractures was 13.6%. A further breakdown of the fracture classification can be seen in Table 4.

**Table 4:** Fracture classification

Type of fracture	Articles reported	*n*	Weighted % of patients
Comminuted	8	216	97.2
Intra-articular	9	241	90.0
Open fracture	6	242	13.6

### Functional outcomes

The functional outcomes are summarised in Table 5. The ROM for extension and flexion (95.7°), pronation and supination (135.9°) and ulnar and radial deviation (28.8°). Grip strength was only documented in three studies and the weighted mean was 71.6% compared to the contralateral side. The weighted mean DASH score averaged 28.6 and the QuickDASH score 19.8.

**Table 5:** Functional outcomes

Functional outcomes	Articles reported	*N*	Weighted mean
DASH	3	80	28.6
Quick DASH	2	39	19.8
Overall DASH	5	119	25.7
Grip strength (%)	3	61	71.6
Flexion	6	144	46.9
Extension	6	144	48.8
Pronation	6	144	68.4
Supination	6	144	67.5
Ulnar deviation	3	68	15.7
Radial deviation	3	68	13.1

### Radiological outcomes

Table 6 shows the accumulated radiological outcomes. The articular step-off was recorded as < 2 mm in all papers, except one patient in Huish et al. [12].

**Table 6:** Radiological outcomes

Radiological outcomes	Articles reported	*n*	Weighted mean
Radial height (mm)	5	114	10.0
Volar tilt (°)	7	210	3.1
Ulnar variance (mm)	6	144	0.5
Radial inclination (°)	6	147	18.8
Articular step-off	1	160	< 2 mm

### Complications

Table 7 is a summary of the different complicate rates. The most common complications were digital stiffness, pain and hardware failure. Overall, the complication rate was 11.4%.

**Table 7:** Complications

	Articles reported	*n*	Rate of complication (%)
Hardware failure	6	278	4.3
Deep infection requiring surgery	7	265	1.9
Superficial infection	7	250	2.4
Total infection (deep and superficial)	9	308	3.6
Symptomatic malunion/non-union	6	280	2.5
Insufficient or loss of reduction	2	46	2.2
Carpel Tunnel decompression	1	24	4.2
CRPS	1	33	3.0
Pain	1	18	11.1
Extensor tendon rupture	8	324	0.6
Extensor tendon lag	2	40	7.5
Tendon adhesions/tenosynovitis	5	231	1.7
Extensor tendon tenolysis with plate removal	4	211	11.4
Digital stiffness	2	55	18.2
Further operation	4	89	5.6
Nerve injury/neuropraxia/neuritis	5	118	2.5
Total			11.4%

### Risk of bias

The risk of bias for each article can be seen in Table 8. All studies had an overall risk of bias of moderate or high. This was mainly due to the confounding factors throughout each of the articles. In addition, due to all studies being retrospective, there was a moderate risk of bias in participation selection and classification of interventions.

**Table 8:** Summary of quality assessment (ROBINS-I)

	Type of bias							Overall risk of bias
	Confounding	Participant selection	Classification of interventions	Deviation from intended intervention	Attrition bias	Detection bias	Reporting bias	
Bouvet et al. [17]	Moderate	Moderate	Moderate	NI	Low	Low	Low	Moderate
Carula et al. [16]	Moderate	Moderate	Moderate	NI	Moderate	Low	Moderate	Moderate
Dodds et al. [15]	Moderate	Moderate	Moderate	NI	Low	Low	Low	Moderate
Hanel et al. [14]	Moderate	Moderate	Moderate	NI	Moderate	Low	Low	Moderate
Hanel et al. [18]	Moderate	Moderate	Moderate	NI	Low	Low	Low	Moderate
Huish et al. [12]	Moderate	Moderate	Moderate	NI	Low	Low	Low	Moderate
Lauder et al. [4]	Moderate	Moderate	Moderate	NI	High	Low	Low	High
Richard et al. [1]	Moderate	Moderate	Moderate	NI	Low	Low	Low	Moderate
Ruch et al. [19]	Moderate	Moderate	Moderate	NI	Moderate	Low	Moderate	Moderate
Sharareh et al. [5]	Moderate	Moderate	Moderate	NI	Moderate	Low	Low	Moderate
Tinsley and Ilyas [13]	Moderate	Moderate	Moderate	NI	Low	Low	Low	Moderate

Multiple studies had incomplete datasets resulting in moderate attrition bias, whilst Lauder et al. [4] had a participation rate of 18% making the attrition bias high risk. The detection bias was low throughout, whereas there was some reporting bias due to gaps in the reported outcomes.

## Discussion

This systematic review evaluates the radiological, functional outcomes and complications associated with DBP fixation of DRFs. Previous systematic reviews have looked at individual outcomes [2, 6], but none looked at a combination of radiological and functional outcomes as well as complications. The literature reveals that radiological outcomes following a DBP are satisfactory, with a complication rate of 11.4%.

Dorsal bridge plates are considered for complex and unstable distal radius fractures. Therefore, these are patients who are expected to have poor outcomes. This review shows that the radiological and functional outcomes are satisfactory for these fracture patterns.

### Radiological outcomes

One concern regarding DBP is the possibility of less satisfactory radiological outcomes compared to volar locking plate fixation, potentially resulting in poorer overall outcomes. However, our review indicates that DBP yields radiological results comparable to those achieved with volar locking plate fixation in AO C2/3 distal radius fractures [20].

Standard radiological parameters such as volar tilt, radial inclination and radial height typically fall within the ranges of 7–15°, 15–25° and 8–14 mm, respectively, with acceptable ranges of < 5° dorsal angulation, > 15 degrees and > 8 mm. In this review, the DBP consistently achieved satisfactory radiological outcomes, within these acceptable ranges (3.1° for volar tilt, 18.8° for radial inclination and 10 mm for radial height). In contrast, fixation with volar locking plate failed to meet the acceptable standard for radial inclination (14.2°), although it did meet requirements for volar tilt (12.5°) and radial height (10.5 mm). Additionally, we observed acceptable articular step-off which was within the range of < 2 mm.

Comparison between DBP and volar locking plate fixation revealed that DBP consistently achieved acceptable radiological outcomes across all parameters. However, whilst volar plate fixation in AO C2/3 fractures met the criteria for volar tilt and radial height, it did not achieve acceptable degrees of radial inclination.

This review suggests that DBP offers a viable option for achieving satisfactory radiological outcomes in complex DRFs, potentially reducing the incidence of malunions. However, it is worth noting that DBP necessitates a second operation for plate removal. Furthermore, it is important to acknowledge that radiological outcomes may not necessarily correlate with functional outcomes, which ultimately determine the patient’s quality of life [6].

### Functional outcomes

As highlighted in Fares et al. [2], the concerns regarding DBP are that prolonged immobilisation leads to a reduced ROM. However, this analysis demonstrates that functional ROM with DBP is significantly improved compared with the use of external fixators, an alternative mode of fixation for these complex fractures, and is comparable to that achieved with volar locking plates.

In this review, the average ROM values for flexion, extension, pronation and supination in patients treated with DBP were 46.9°, 48.8°, 68.4° and 67.5°, respectively. In comparison, Oner et al. reported improved flexion, extension and supination (79.5°, 62.8° and 71.7°, respectively) in the use of volar locking plates in AO C2/3 fractures; however, there was a reduction in pronation (64.4°). Despite this, the results indicate that a functional arc of 95.7° can be achieved with DBP fixation, which exceeds the range for most activities of daily living.

Regarding patient-reported outcomes, the DASH score at final follow-up was 25.1, measured between 19.5- and 150.8-week post procedure. However, it is important to note that Ruch et al. [19] reported a decrease in DASH score from 33.8 at 6 months to 11.5 at 2 years, suggesting that our DASH score may not fully capture the long-term outcomes of DBP [19]. In contrast, Oner et al. [20] reported a Quick DASH score of 4.94 at 1-year post-volar locking plate fixation [20]. Similarly, the grip strength with a volar plate was found to have a 10.4% loss compared to a 28.4% loss with DBP.

In summary, whilst DBP demonstrates acceptable functional outcomes, they are still inferior to volar locking plates.

### Complications

In this review, the overall rate of complications associated with DBP was 11.4%, which is notably lower compared to reported complication rates of up to 23% for volar plating [21] and 63% for external fixation [22]. The most frequently encountered complications included digital stiffness, pain, hardware failure and infection.

An analysis by Hanel et al. [18] found that the complication rate was influenced by the duration the plate remained implanted. Patients who retained the plate for more than 16 weeks experienced a complication rate of 20.8%, whereas those who underwent plate removal earlier had a lower rate of 8.5% [18]. In this review, the mean time to plate removal in this review was 17.3 weeks.

Although the incidence of digital stiffness with DBP was relatively high at 18.2%, it was substantially lower than observed with external fixators [16]. Rates of digital stiffness improved when tenolysis was performed during plate removal [15].

Hardware failure was only observed in patients who retained the plate for longer than 6 months, after the fracture has healed [11, 15], indicating a need for plate removal. Additionally, the choice of plate for fixation plays a crucial role. Studies have shown that the 2.4-mm plate may not adequately support the axial weight bearing in polytrauma patients, potentially leading to early hardware failure. In cases where early weight bearing is needed, consideration should be given to using a 3.5-mm DBP [23].

The complications rates associated with DBP were significantly lower than those observed with volar plating in C-type fractures, as well as external fixators. Sharareh et al. [5] reported an overall complication rate of 11.3% for volar plates, with rates as high as 20% in C-type fractures. Whilst this review did not specifically analyse complication rates by fracture classification, these findings support the use of DBP in C-type fractures [5].

## Limitations

This systematic review is limited by the number of papers and consequently the number of patients analysed. Currently, there is a sparsity of high-level studies in the literature, with only retrospective analyses and case studies available. These study types have inherent limitations such as: loss to follow-up, inconsistent evaluation of the functional and radiological outcomes and potential bias introduced by operating surgeons reporting the results during follow-up. In addition, the subjective interpretation of ROM further adds to the complexity of assessing outcomes [18].

The follow-up periods in the included studies were relatively short, with only two studies extending beyond 100 weeks [4, 19], while the remainder were less than 55 weeks. Consequently, the long-term effects, as well as the functional and radiological outcomes associated with DBP, remain uncertain. Additionally, the heterogeneity of outcomes in this review may have introduced interpretation bias.

The fact that five out of the 11 studies share authors suggest a limited pool of research available and raise concerns about potential bias. Previous systematic reviews have also included a study by Jain et al. who have since been retracted due to methodological flaws [24]. Furthermore, some of these reviews incorporated papers consisting of only one or two case studies, heightening the risk of bias.

To mitigate these limitations and provide more robust evidence, a randomised control trial comparing all three techniques (volar plating, DBP and external fixation) is warranted. Such a trial would not only help to minimise bias but also offer more reliable insights into the comparative effectiveness of these approaches.

## Conclusion

Despite the small cohort of patients, this review has shown that DBP has been used with adequate outcomes, comparable in radiological and functional outcomes to current fixation methods and a significantly lower complication rate than external fixation. Further research with meta-analysis in the future is needed. The DBP should be considered by clinicians as an option for select patients and for patients with complex fractures.
